# Eribulin monotherapy improved survivals in patients with ER-positive HER2-negative metastatic breast cancer in the real world: a single institutional review

**DOI:** 10.1186/s40064-015-1422-8

**Published:** 2015-10-19

**Authors:** Junichiro Watanabe

**Affiliations:** Breast Oncology, Shizuoka Cancer Center Hospital, 1007 Shimonagakubo, Nagaizumi-cho, Sunto-gun, Shizuoka, 411-8777 Japan

**Keywords:** Chemotherapy, Eribulin mesylate, Japanese, Metastatic breast cancer

## Abstract

Despite being routinely prescribed worldwide for several years, data regarding the safety, efficacy, and survival benefit of eribulin in clinical settings for the treatment of metastatic breast cancer (MBC) are limited. This retrospective observational study investigated the survival benefit of eribulin compared with conventional chemotherapy regimens in Japanese women with MBC. Women with estrogen receptor (ER)-positive human epidermal growth factor receptor 2 (HER2)-negative (ER+/HER2−) MBC, including unresectable locally advanced breast cancer, treated at a single institution were included in this study. The primary efficacy measure assessed overall survival (OS), and safety was evaluated as the number of grade 3 and 4 adverse events. Of the 293 patients analyzed, 66 received eribulin (eribulin arm) and 227 received conventional chemotherapeutic agents excluding eribulin (noneribulin arm). The median OS from MBC diagnosis in the eribulin arm was 72.1 months (95 % CI 13.3–168.3) compared with 43.3 months (95 % CI 9.1–202.0) in the noneribulin arm [hazard ratio (HR): 0.67, 95 % CI 0.47–0.96; *P* = 0.025]. No significant differences were noted in OS between eribulin used as a first-/second-line or third-/>third-line treatment for MBC. No patient discontinued eribulin therapy due to AEs. In the eribulin arm, grade 4 neutropenia and grade 3 febrile neutropenia were observed in 8 (12.1 %) and 4 (6.1 %) patients, respectively. Eribulin therapy has a survival benefit in Japanese women with ER+/HER2− MBC in routine clinical practice, with no unexpected grade 3/4 AEs. Interestingly, eribulin might be beneficial as any line therapy for ER+/HER2− MBC.

## Background

The treatment of metastatic breast cancer (MBC) remains a challenge, with approximately 6–10 % of new breast cancer cases being initially diagnosed as stage IV or metastatic in nature (Cardoso et al. 2011). The current therapies generally focus on symptomatic control and prolonging survival. Despite the development of novel treatment options, the improvement of long-term survival in women with MBC still remains a challenge. The 5-year relative survival is reportedly as low as 24.3 % for patients with distant metastases (Howlader et al. 2013), warranting effective treatment options with an increased survival benefit. Although systemic neo-adjuvant/adjuvant chemotherapy may reduce mortality risks in those with breast cancer (Berry et al. 2005), the choice of chemotherapy regimen might affect long-term outcomes. Anthracycline- and taxane-based regimens remain the standard chemotherapy options for neo-adjuvant/adjuvant and/or first-line treatment for MBC; however, disease progression is often attributed to primary or acquired resistance to these regimens. Subsequently, few therapeutic options are available for patients with anthracycline- and taxane-resistant or refractory MBC. Despite limited evidence, these active chemotherapies are listed as potential sequential therapies in late-line treatment (Cardoso et al. 2014; National Comprehensive Cancer Network. NCCN clinical practice guidelines in oncology breast cancer, version 2 2015).

Recently, eribulin, a nontaxane microtubule dynamics inhibitor belonging to the halichondrin class of antineoplastic agents, has shown anticancer activity in women with MBC. Three phase II studies have demonstrated that eribulin has potent anticancer activity with acceptable tolerability (Aogi et al. 2012; Cortes et al. 2010; Vahdat et al. 2009). A phase III randomized study (study 305, NCT00388726 or EMBRACE) demonstrated a significant and clinically meaningful improvement in survival with eribulin compared with the physician’s choice of treatment in women who were heavily pretreated with manageable toxicity (Cortes et al. 2011). In another phase III randomized study (study 301), eribulin showed a favorable improvement in overall survival (OS) compared with capecitabine; however, this improvement did not meet the predefined criteria for statistical significance (Kaufman et al. 2015. In addition, a pooled analysis of both the aforementioned phase III studies demonstrated that eribulin-treated patients had a significantly prolonged OS compared with the controls. Moreover, the OS results for eribulin were favorable in all population subgroups (Twelves et al. 2014). Based on the results of these studies, eribulin has been approved in the United States and certain other countries for the treatment of MBC previously treated with at least two chemotherapeutic agents, including anthracycline- and taxane-based regimens. In Europe, eribulin has been approved for the treatment of MBC previously treated with at least one chemotherapy regimen. In Japan, on the other hand, eribulin has been approved for the treatment of patients with inoperable or recurrent breast cancer but not limited to those who have been previously treated with chemotherapy regimens for MBC.A phase II study of eribulin in Japanese patients with locally advanced disease or MBC who were hormone receptor and HER2 positive as well as previously treated with an anthracycline and a taxane, eribulin demonstrated an OS of 11.1 months, respectively (Aogi et al. 2012).

Several retrospective and prospective studies have demonstrated the therapeutic benefits of eribulin (Aogi et al. 2012; Cortes et al. 2010; Vahdat et al. 2009; Cortes et al. 2011; Kaufman et al. 2015; Twelves et al. 2014; Gamucci et al. 2014; Poletti et al. 2014); however, extrapolation of these findings to real-world settings remains to be explored. Although eribulin has been clinically used as a treatment option in Japanese women diagnosed with MBC for over a year, evidence of its safety and efficacy in clinical settings is not extensively documented. Moreover, the survival benefit of eribulin over conventional chemotherapy is yet to be determined; thus, the present study was conducted to investigate the survival benefit of eribulin compared with conventional chemotherapy regimens in Japanese women with MBC in routine clinical practice.

## Results

### Patients

A total of 325 patients with ER+/HER2− MBC including LABC were assessed in this study. Of these, 293 patients (214 with metastatic and 79 with locally advanced disease) had been treated with at least one chemotherapy regimen recommended by the NCCN guidelines for the treatment of MBC (eribulin arm: 66 patients; noneribulin arm: 227 patients). Baseline patient characteristics at diagnosis of MBC are presented in Table 1. The median age in both eribulin and noneribulin arms was 54.0 years (range 27–77 and 25–78 years, respectively). In the eribulin arm, the median exposure to eribulin was 4.2 months, and the median relative dose intensity was 47.4 %. The median duration of eribulin treatment was 125 days, ranging from 2 to 454 days. All the patients had stage IV disease and an ECOG PS 0–2.

**Table 1:** Baseline demographics and clinical characteristics at diagnosis of metastatic breast cancer

Variables	Overall		Eribulin		Noneribulin		P value Fisher’s exact test
	(N = 293)		(N = 66)		(N = 227)		
Median age, years (range)	54.0 (25–78)		55.0 (27–77)		54.0 (25–78)		
Diagnosis, n (%)							
Metastatic	214	(73.0)	44	(66.7)	170	(74.9)	0.21
Locally advanced	79	(27.0)	22	(33.3)	57	(25.1)	
Sites of metastases, n (%)							
Lung	71	(24.2)	16	(24.2)	55	(24.2)	1.00
Liver	58	(19.8)	20	(30.3)	38	(16.7)	0.02*
Bone	155	(52.9)	35	(53.0)	120	(52.9)	1.00
CNS	14	(4.8)	3	(0.5)	11	(4.8)	1.00
Soft tissue	185	(63.2)	44	(66.7)	141	(62.1)	0.56
Treatment regimens, n (%)							
Anthracycline-based regimen for EBC	79	(27.0)	22	(33.3)	57	(25.1)	
Anthracycline-based regimen for MBC	135	(46.1)	26	(39.4)	109	(48.0)	
Taxane-based regimen for EBC	39	(13.3)	18	(27.3)	21	(9.3)	
Taxane-based regimen for MBC	228	(77.8)	53	(80.3)	175	(77.1)	
Eribulin as first-/second-line for MBC	31	(47.0)	31	(47.0)	–		
Taxane-based regimen prior to eribulin	40	(13.7)	40	(60.6)	–		

*CNS* central nervous system, *EBC* early breast cancer, *MBC* metastatic breast cancer

### Efficacy outcomes

**Fig. 1:**
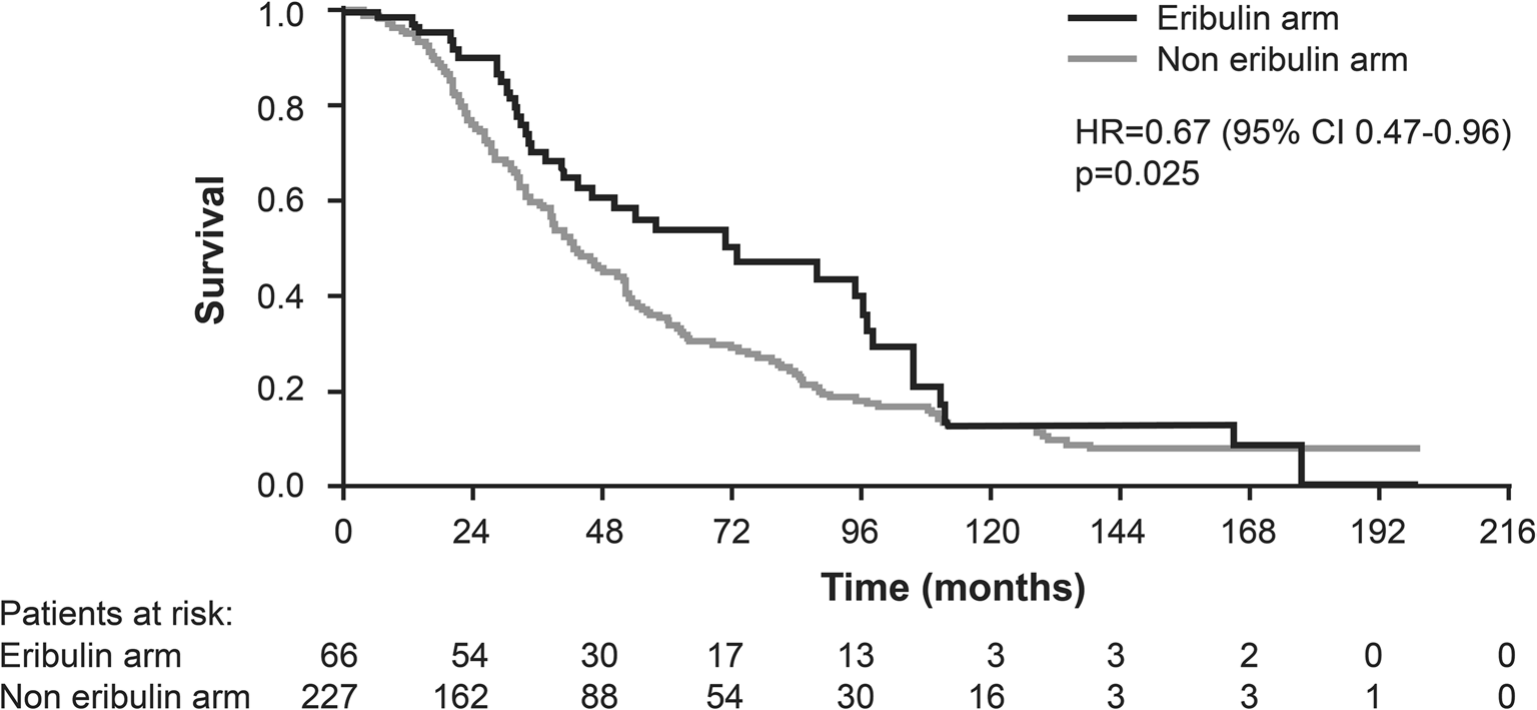
Kaplan–Meier estimates of overall survival. The primary efficacy measure in the present study assessed overall survival, which was calculated as the time from diagnosis of metastatic breast cancer until death from any cause. HR denotes hazard ratio for the eribulin arm as compared with the noneribulin arm

A total of 214 (73.0 %) patients died during the study period, of whom, 39 were from the eribulin arm and 175 from the noneribulin arm. Kaplan–Meier survival curves for OS in the eribulin and noneribulin arms are shown in Fig. 1. The median OS from diagnosis of MBC was 72.1 months (95 % CI 13.3–168.3) in the eribulin arm compared with 43.3 months (95 % CI 9.1–202.0) in the noneribulin arm; OS was significantly prolonged in the eribulin arm (HR: 0.67, 95 % CI 0.47–0.96; *P* = 0.025).

The influence of chemotherapy regimens on OS was analyzed using a Cox proportional hazards model, controlling for each factor at a time. Based on the analyses, only eribulin monotherapy significantly prolonged the OS (HR: 0.55, 95 % CI 0.36–0.85; *P* = 0.01; Table 2). In contrast, patients who received an anthracycline-based regimen (HR: 1.35, 95 % CI 1.01–1.82; *P* = 0.04) or a paclitaxel + gemcitabine combination regimen (HR: 2.79, 95 % CI 1.34–5.79; *P* = 0.01) showed significantly poor outcomes (Table 2).

**Table 2:** Administered agents/regimens and hazard ratio for overall survival

Agents/regimens	No. of patients	No. of events, n (%)		HR	95 % CI	*P* value
Eribulin monotherapy	66	39 (59.1)		0.55	0.36–0.85	0.01*
Anthracycline-based regimen	136	120 (88.2)		1.35	1.01–1.82	0.04*
Paclitaxel monotherapy	138	103 (74.6)		1.14	0.85–1.52	0.38
Docetaxel monotherapy	79	72 (91.1)		1.30	0.95–1.79	0.10
Capecitabine monotherapy	120	100 (83.3)		0.89	0.66–1.20	0.43
Gemcitabine monotherapy	12	12 (83.3)		0.55	0.27–1.10	0.09
Vinorelbine monotherapy	37	37 (100.0)		1.15	0.76–1.73	0.51
Paclitaxel + bevacizumab	52	52 (57.7)		1.51	0.93–2.48	0.10
Docetaxel + capecitabine	30	30 (96.7)		1.13	0.74–1.73	0.57
Paclitaxel + gemcitabine	10	10 (100.0)		2.79	1.34–5.79	0.01*

*CI* confidence interval, *HR* hazard ratio

Furthermore, among the patients who were treated with eribulin, the influences of clinical characteristics of the patients and specific treatments at initiation of eribulin on OS were analyzed. Prior use of an anthracycline-based regimen and duration of treatment with eribulin were statistically significant clinical variables associated with prolonged OS in MBC (HR: 0.36, 95 % CI 0.11–0.83; *P* = 0.02 and HR: 0.45, 95 % CI 0.21–0.94; *P* = 0.032; Table 3). No significant differences were observed in OS with the use of eribulin as first-, second-, third-, or beyond third-line treatment for MBC.

**Table 3:** Prognostic factors for overall survival in the eribulin arm

Variables	n	HR	95 % CI	*P* value
Lung metastasis	27	0.90	0.42–1.95	0.79
Liver metastasis	36	1.25	0.56–2.78	0.59
Bone metastasis	43	1.42	0.58–3.50	0.44
CNS metastasis	9	1.36	0.44–4.17	0.59
Other metastasis	47	0.46	0.20–1.02	0.06
Anthracycline-based regimen for EBC	21	0.51	0.15–1.73	0.28
Anthracycline-based regimen for MBC	24	0.36	0.11–0.83	0.02*
Taxane-based regimen for EBC	13	0.48	0.08–2.77	0.41
Taxane-based regimen for MBC	41	0.66	0.22–1.96	0.45
Eribulin as first-/second-line for MBC	31	0.90	0.37–2.20	0.82
Taxane-based regimen prior to eribulin	28	1.41	0.63–3.14	0.40
Duration of eribulin treatment >median (125 days)	33	0.45	0.21–0.94	0.03*

*CI* confidence interval, *CNS* central nervous system, *EBC* early breast cancer, *HR* hazard ratio, *MBC* metastatic breast cancer

### Safety outcomes

No patient discontinued eribulin therapy due to adverse events. No grade 3 or 4 neuropathy was documented. Grade 4 neutropenia was observed in 8 (12.1 %) patients, and grade 3 febrile neutropenia was observed in 4 (6.1 %) patients. None of these events was related to eribulin according to the investigator. No other grade 3/4 hematological or non-hematological toxicity was documented.

## Discussion

This retrospective observational study investigated the survival benefit of eribulin compared with other conventional chemotherapy regimens in Japanese women with ER+/HER2− MBC. The present analyses revealed a prolonged OS in patients treated with eribulin than in those treated with other chemotherapy regimens. In addition, these analyses showed that eribulin monotherapy provided a survival benefit with an efficacy that is independent of its use as a front- or late-line therapy. Moreover, no patient discontinued eribulin therapy due to adverse events.

The median OS for eribulin-treated patients was 72.1 months, suggesting that the median OS recorded in this study is significant, particularly in patients who had been treated with eribulin for more than 125 days. Currently, no treatment standard has been established for patients with MBC requiring third- or fourth-line therapy. The results of the EMBRACE study (Cortes et al. 2011) and the pooled analysis of two phase III studies (EMBRACE and study 301) (Twelves et al. 2014) suggest that eribulin could prolong survival in patients with late-stage MBC, particularly in those who have been heavily pretreated. In the present study, eribulin demonstrated a significant survival benefit in patients treated with an anthracycline-based regimen for MBC; thus, eribulin may be beneficial for patients who did not respond to an anthracycline-based regimen. In addition, almost half of the eribulin-treated patients had received the drug as a third- or beyond third-line treatment, and the efficacy of eribulin in these patients was comparable to those who received eribulin as first- or second-line treatment. Taken together, eribulin might be beneficial not only as a late-line but also as a front-line treatment for MBC. In certain countries, eribulin has been approved for the treatment of patients with MBC who were previously treated with at least two chemotherapy regimens, including anthracycline- and taxane-based regimens; however, in Japan, eribulin has been approved for patients with inoperable or recurrent breast cancer, irrespective of their previous treatment history. The present study supports the indication of eribulin use in Japan and demonstrates its potential as a therapeutic option for patients with MBC, regardless of the treatment line.

Since HER2 has been shown to play a vital role in the development and progression of certain types of breast cancer, several novel anti-HER2 therapeutic agents, including trastuzumab, lapatinib, pertuzumab, and trastuzumab emtansine have been developed. Based on the Surveillance, Epidemiology, and End Results registries in the United States, 72.7 % patients with MBC were found to be hormone receptor positive (HR+)/HER2−, and 12.2 % were triple-negative (HR−/HER2−) (Howlader et al. 2014); however, the aforementioned novel anti-HER2 therapeutic agents may not be suitable for a majority of these patients. Compared with diseases that exhibit an HER2-positive profile, those with an HER2-negative profile are heterogeneous, and biomarkers for such diseases have not yet been established. Thus, additional investigations on the benefits of treatment independently with biomarkers are warranted. According to the results of the present study, the survival benefit of eribulin was observed among the patients with ER+/HER2− MBC. A subgroup analysis of the previous pooled analysis showed that eribulin has a tendency to prolong OS which was not statistically significantly in the subpopulation with ER+/HER2− MBC (HR: 0.86, 95 % CI 0.74–1.01; *P* = 0.06) (Twelves et al. 2014). These study results support the use of eribulin as a potential therapeutic option for the treatment of ER+/HER2− MBC; however, additional investigations on the benefits of eribulin in such patients are warranted.

In the present study, eribulin had an acceptable safety profile. Grade 3 and 4 neutropenia were observed in 6.1 and 12.1 % patients, respectively, and no other grade 3/4 hematological or non-hematological toxicity was documented. Notably, grade 3/4 peripheral neuropathy, the most common adverse event leading to discontinuation of eribulin therapy in EMBRACE, was not observed in the present study. In addition, no patient discontinued eribulin therapy due to adverse events. Based on the low incidence of adverse events and no treatment discontinuation, the safety profile of eribulin in the present study may be considered more acceptable than that in previously reported phase II/III studies (Aogi et al. 2012; Cortes et al. 2010; Vahdat et al. 2009; Cortes et al. 2011; Kaufman et al. 2015; Twelves et al. 2014). The median relative dose intensity of eribulin was 47.4 %, and the dose was modified to prevent the occurrence of unwarranted toxicity at the physician’s discretion. Hence, the dose of eribulin may be modified based on an individual patient’s needs to minimize its toxicity and maximize benefits.

Unlike randomized clinical trials that have strict inclusion criteria, the retrospective nature of this study allowed the inclusion of a variety of patients. Since patient groups are usually more heterogeneous, these findings may provide a realistic picture of what is observed in routine clinical practice as the strength of the study. However, the present study has a few limitations. This was a retrospective observational study, and the two arms were compared without randomization; thus, interpretation and generalization of the findings should be conducted with caution. The sample size was relatively small, and this was an exploratory study and not a confirmatory one. In addition, subjective bias may have had some impact on the results.

## Conclusion

In summary, as with phase II/III studies, the present study which retrospectively investigated the efficacy and safety of eribulin in Japanese women with ER+/HER2− MBC in routine clinical practice demonstrated a significant survival benefit of eribulin in such patients, with a relatively lower dose intensity and no unexpected grade 3 and 4 adverse events. The survival benefit of eribulin was regardless of the organs involved, previous treatment regimens, or line of treatment. Eribulin might be beneficial as any line treatment for ER+/HER2− MBC; however, further validation of the current findings should be performed in a larger study.

## Methods

### Patients

This retrospective observational study included Japanese women with MBC, including unresectable locally advanced breast cancer (LABC), who were treated at the Shizuoka Cancer Center Hospital (Shizuoka, Japan) between October 1, 2002, and November 30, 2014. Women diagnosed with ER-positive HER2-negative (ER+/HER2−) MBC and treated with at least one chemotherapy regimen were included. Eribulin was administered based on the approved dosage and administration practice in Japan, via drip intravenous infusion at a maximum flow of 1.4 mg/m^2^ on days 1 and 8 every 3 weeks. Other conventional chemotherapeutic agents were also administered based on the approved dosage and administration practices in Japan. Dose modifications such as dose reduction or dose omission on day 8 were permitted based on the physician’s discretion. Treatment could be discontinued based on the physician’s decision (e.g., disease progression, unacceptable toxicity, or worsening health status) or the patient’s request to do so.

The study design was approved by the Clinical Research Ethics Committee and the study was conducted in accordance with the latest Ethical Guidelines for Medical and Health Research Involving Human Subjects and the local regulations in Japan.

### Outcome assessments

Demographics and baseline characteristics of all the patients, including age, HER2 and ER status, treatment history, and sites of metastases were collected upon diagnosis of MBC. In addition, the median treatment duration and dose intensity of eribulin were assessed. The primary efficacy measure assessed OS, which was calculated as the time from the diagnosis of MBC until death from any cause. To evaluate safety, grade 4 neutropenia, grade 3 febrile neutropenia, and grade 3/4 hematological or non-hematological toxicity were assessed only in patients who received eribulin. Adverse events were recorded and graded according to the National Cancer Institute Common Terminology Criteria for Adverse Events (version 3.0, Japanese version).

### Statistical analysis

The final data cut-off date was November 30, 2014. OS curves for patients who received eribulin (eribulin arm) and conventional chemotherapy excluding eribulin (noneribulin arm) were estimated using the Kaplan–Meier method, and both arms were compared using the log-rank test. Hazard ratios (HRs) and 95 % confidence intervals (CIs) were estimated using the Cox proportional hazards model to investigate the influence of chemotherapy regimens on OS. Moreover, subgroup analyses were performed using the Cox proportional hazards model to identify clinically relevant interactions between treatment outcomes and prognostic factors including sites of metastases, previous treatments, duration of treatment, and the time of initiation of eribulin. For multivariate analyses, patients who were treated with agents not recommended according to the latest National Comprehensive Cancer Network (NCCN) guidelines (National Comprehensive Cancer Network. NCCN clinical practice guidelines in oncology breast cancer, version 2 2015) (e.g., TS-1 monotherapy and capecitabine + cyclophosphamide combination therapy) were excluded owing to the small number of patients. All statistical analyses were two-sided, and *P* values <0.05 were considered statistically significant.

## Abbreviations

ER+/HER2−: estrogen receptor (ER)-positive human epidermal growth factor receptor 2 (HER2)-negative
HR: hazard ratio
LABC: locally advanced breast cancer
MBC: metastatic breast cancer
OS: overall survival
